# Synergy of Ionic and Dipolar Effects by Molecular Design for pH Sensing beyond the Nernstian Limit

**DOI:** 10.1002/advs.201901001

**Published:** 2019-11-27

**Authors:** Chiao‐Wei Tseng, Chenyu Wen, Ding‐Chi Huang, Chin‐Hung Lai, Si Chen, Qitao Hu, Xi Chen, Xingxing Xu, Shi‐Li Zhang, Yu‐Tai Tao, Zhen Zhang

**Affiliations:** ^1^ Division of Solid‐State Electronics The Ångström Laboratory Uppsala University SE‐751 21 Uppsala Sweden; ^2^ Institute of Chemistry Academia Sinica Taipei 115 Taiwan; ^3^ Department of Medical Applied Chemistry Chung Shan Medical University Taichung 40201 Taiwan

**Keywords:** dipole moments, ion‐sensitive field‐effect transistors, pH sensitivity, super‐Nernstian response, surface functionalization

## Abstract

Knowledge of interfacial interactions between analytes and functionalized sensor surfaces, from where the signal originates, is key to the development and application of electronic sensors. The present work explores the tunability of pH sensitivity by the synergy of surface charge and molecular dipole moment induced by interfacial proton interactions. This synergy is demonstrated on a silicon‐nanoribbon field‐effect transistor (SiNR‐FET) by functionalizing the sensor surface with properly designed chromophore molecules. The chromophore molecules can interact with protons and lead to appreciable changes in interface dipole moment as well as in surface charge state. In addition, the dipole moment can be tuned not only by the substituent on the chromophore but also by the anion in the electrolyte interacting with the protonated chromophore. By designing surface molecules to enhance the surface dipole moment upon protonation, an above‐Nernstian pH sensitivity is achieved on the SiNR‐FET sensor. This finding may bring an innovative strategy for tailoring the sensitivity of the SiNR‐FET‐based pH sensor toward a wide range of applications.

## Graphical abstract

Synergy of surface charges and dipole changes induced by interfacial protons interactions enables potential response of sensors based on nanoribbon field‐effect transistors beyond the Nernstian limit. Benefiting from tunability of the dipole effect not only by controlling the molecular substituents (i.e., molecular dipole) but also by modulating the electrolyte anions (i.e., ionic dipole), artificially controllable sensing response can be achieved.

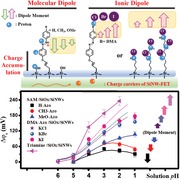

Development of high sensitivity electronic sensors has attracted tremendous interest to meet urgent demands on portable devices for healthcare diagnostics and environmental monitoring.^1^, ^2^ In a common liquid sensing system, interaction between analytes and functional receptors at the liquid/solid interface generates original signal and plays a crucial role in the sensing process. In ion sensors, the interaction between ions and ion receptors can lead to accumulation of interfacial charges, thereby causing an interfacial potential change that can be electronically readout.^3^ A versatile sensing platform can also be designed by functionalizing the sensor surface with self‐assembled organic monolayers (SAMs), which offers the possibility to tailor the chemical and/or physical interactions of the sensor surface with analytes. These interfacial interactions can generate additional changes in surface dipoles and/or charges on the sensing surface,^4^, ^5^ which can in turn affect the electrical properties of the surface and tune the sensitivity.^6^, ^7^, ^8^ It can be emphasized that both surface charge and dipole play an equally important role in establishing the surface equilibrium state. The dipole effect can be engineered in a controllable and systematic way. Therefore, modulating the molecular dipole moment to enhance device performance holds promises in numerous applications.^9^, ^10^, ^11^ However, the molecular dipole effect is strongly dependent on multiple parameters in practice, such as dipole orientation, distribution, and packing density of each molecule within the SAM.^12^, ^13^ These parameters are strongly inter‐correlated. For example, increasing the dipole moment of the surface immobilized molecules can enhance the electrostatic repulsions between neighboring dipoles, influence the packing density, and even change the configuration of the SAM. Thus, in order to engineer the interfacial dipole in a controllable manner, a rational design of the system is needed.

Since the dipole moment originates from the charge separation between atoms due to their difference in electronegativity, changing the electronegativity on the molecular substituent can be an efficient way to modulate the molecular dipole. A dipole moment can also be formed by ion pair of cation and anion,^14^, ^15^ which can be embodied by a well‐known phenomenon that the electrostatic attraction between cations and anions can lead to a significant change in surface wettability.^16^, ^17^ This ionic dipole is strongly dependent on the polarizability of the ionic pair, and is affected by the properties of the ions such as ionic radius and hydration energy.^18^, ^19^ Therefore, artificial designs to form ion pairs can be an alternative to create dipoles on the sensing surface for modulation of its sensitivity. With proper molecular engineering, both approaches to form dipoles can be realized via molecule–analyte interactions to tailor the sensitivities in various sensing applications. Even though the effect of surface dipole has been known and employed to tailor the properties of different substrates, there is still a lack of systematic study on utilizing the dipoles with controlled direction and magnitude for sensing applications through rational design of the SAMs.

In this work, artificial tailoring of the sensitivity via the dipole moment of the SAMs was demonstrated on a silicon‐nanoribbon field‐effect transistor (SiNR‐FET)‐based pH sensor. The silicon FET was selected for its well‐known superior reliability comparing to FET devices made of other channel materials. In addition, it has been shown to be sensitive to surface potential variations associated with the interaction occurring at the sensing interface, and it is hence a suitable platform to investigate ion–surface interactions.^20^, ^21^ pH value is an important parameter in numerous fields. Almost every biological process is pH‐dependent, especially in the range from 8 to 3, where the pH variations reflect a myriad of critical information such as human health,^22^, ^23^ viral behavior,^24^, ^25^ bacterial activity,^26^ and so on. Therefore, developing an ultrasensitive pH sensor has extremely high practical importance.^27^, ^28^ Here, a polarizable azobenzene (azo) chromophore was employed to impose a dipole moiety via the protonation process of molecule.^29^ Azo is a classic pH indicator dye,^30^, ^31^ but its application is severely limited due to its poor water solubility and serious aggregations after protonation.^32^, ^33^ In our demonstration, the azo molecules were directly anchored on the sensor surface (SiO_2_) to avoid the solubility and aggregation issues. Protonation of the azo molecules will generate a dipole moment change on the sensor surface. In addition to the dipole effect, the sensor surface has two types of active sites which can interact physically with protons in electrolyte, i.e., the azo groups in the SAM and the hydroxyl groups on the SiO_2_ surface. Furthermore, the dipole effect imposed by the azo chromophore can be generated from either the molecular dipole in the chromophore molecules or the ionic dipole between the protonated chromophore substituent and the anions in the electrolyte. Therefore, the interfacial dipole moment can be tuned not only by the substituent on chromophores with different electron‐withdrawing or electron‐donating power but also by the anion in the electrolyte. In this paper, we employed different substituents (CF_3_, CH_3_, CH_3_O (i.e., MeO), and (CH_3_)_2_N (i.e., dimethylamine (DMA)) with their electron‐donating power in the order CF_3_ < CH_3_ < MeO < DMA), as well as different electrolyte anions (Cl^−^, Br^−^, and I^−^) to tune the polarizability of chromophore molecules and the ionic pair, i.e., protonated chromophore‐anion pair. With the synergy effects of dipole moment change and charge accumulation on the sensor surface, a super Nernstian pH sensitivity with an over 200% increase of pH sensitivity in the pH range 5–3 comparing to that of a commonly used sensor with a bare SiO_2_ sensing layer (12 to 36 mV pH^−1^) was achieved. In general, we demonstrate a novel strategy to manipulate the sensitivity by tailoring surface dipole moments.

A series of molecules with different substitutes is carefully designed, which can interact with protons and act as the sensitive layer to functionalize on the sensor's surface. To elucidate the substituent effect on protonation and the dipole moment of the azo chromophores, electronic absorption spectroscopy (UV–vis) analysis and density functional theory (DFT)‐based calculations were employed to characterize the chromophore. The chemical structures and corresponding abbreviations of the merged molecules, the azobenzene (azo)‐carrying silanes, are shown in **Figure** **1**a. Details regarding the synthetic procedures and structure characterization of azo chromophores are shown in the Supporting Information. Protonation of azo chromophores (H‐Azo) was recorded by the UV–vis absorption spectroscopy, as shown in the inset of Figure 1b and more spectra for azo chromophores with substituents can be found in Figure S3 (Supporting Information). Owing to the poor solubility of the azo chromophores in water, the measurements were carried out in dichloromethane with the solution pH adjusted with trifluoroacetic acid. At lower pH, i.e., pH < 4, a new band at longer wavelength (≈480 nm) was appeared at the expense of the original π–π* transition band of the azo moiety (≈350 nm), suggesting the formation of the protonated azo, azonium ion.^34^ Normalized absorbance of the azonium band as a function of solution pH for all the chromophore studied was summarized in Figure 1b. It is noted that the amount of azonium ion is dependent not only upon the pH but also on the chromophore substituent. Except for the CF_3_‐ and N(CH_3_)_2_ (DMA)‐substituted azo, the other azo chromophores were protonated at around pH 3. The CF_3_‐substituted azo (CF_3_‐Azo) starts protonation at around pH 1–2 as it is more difficult to be protonated, while the protonation of DMA‐substituted azo (DMA‐Azo) was started at around pH 4–5 as it is easier to be protonated, in comparison to other azo chromophores. Direct comparison of the absorbance data suggests a clear substituent effect. The stronger donor substituent (such as DMA) results in a much stronger basicity of the azo moiety, which leads to an enhanced protonation to form the azonium band.^35^, ^36^ On the other hand, the strong electron‐withdrawing substituent (such as CF_3_) reduces the basicity of the chromophore and thus hinders the protonation process.

**Figure 1:**
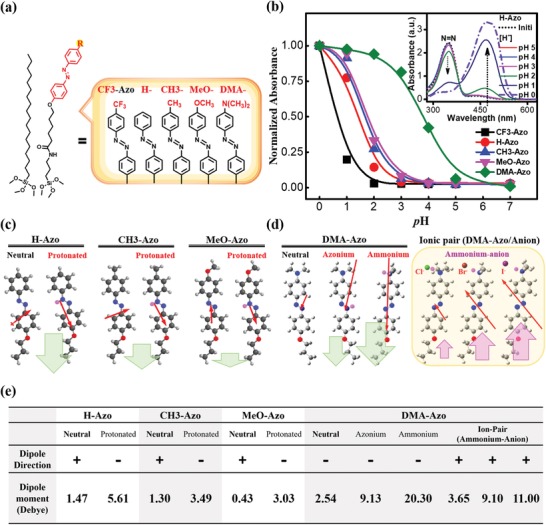
a) Structures and abbreviations of azo chromophores used in this study. b) UV–vis absorption spectra. (Inset: absorption spectra of H‐Azo chromophore in different pH solutions.) The relative intensity of the emerged absorption peak (between 450 and 600 nm) is shown as a function of pH for all azo chromophores used in this work. The intensity was extracted from the spectra and normalized to the peak intensity at pH = 0. c) DFT calculations of dipole moments for: H‐Azo, CH_3_‐Azo, and MeO‐Azo at neutral and protonated states. d) Left: DFT calculations of dipole moment for DMA‐Azo at neutral and two protonated states, i.e., the azonium (protonated N=N) and the ammonium (protonated DMA substituent) ions. Right: DFT calculations of dipole moments for the ion‐pairs formed between the protonated chromophore (ammonium ion) and the halide anions (Cl^−^, Br^−^, and I^−^). For all azo chromophores, the colored balls on the molecular structure represent different atoms: black (carbon), gray (hydrogen), red (oxygen), blue (nitrogen), and pink (hydrogen from protonation). The red arrows show the amplitude and direction of dipoles according to DFT calculations and the corresponding values are listed at the table below. The green and pink arrows illustrate the dipole moment changes for each case in comparison to its neutral case. e) The table shows the value and direction of dipoles based on DFT calculations. The positive and negative symbols represent the dipole with positive and negative charges on the bottom of structures, respectively.

In addition, DFT‐based calculations were carried out to gain insight into the dipole moment of the azo chromophores at neutral and protonated states, as shown in Figure 1c,d. The dipole variation of the CF_3_‐Azo chromophore was not shown here since it is too difficult to be protonated according to the UV–vis data. Details regarding the DFT calculations are shown in the Supporting Information. Herein, the nucleophilicity of the active atoms of the chromophore and the Gibbs free energy (Δ*G*) of the corresponding protonated species were adopted to determine the protonated structures, as listed in Table S1 (Supporting Information). The protonated azo (H‐Azo), azonium ion, has an opposite dipole direction comparing to the neutral chromophore (H‐Azo) before protonation. The difference in dipole moments between the neutral and protonated species strongly depends on the substituent of the chromophore. As shown in Figure 1c, the MeO‐substituted azo (MeO‐Azo) has the smallest change in dipole moment (3.46 D) upon protonation compared to the other substituted azo chromophores (7.08 and 4.79 D for H‐Azo and CH_3_‐Azo, respectively). It is evident that the change in dipole moment of H‐Azo, CH_3_‐Azo, and MeO‐Azo chromophores during protonation decreases for azo with increasing electron‐donating power (note that the electron‐donating power: H‐Azo < CH_3_‐Azo < MeO‐Azo). However, the DMA‐Azo case is more complicated due to the strong basicity of its amine group. As shown in Figure 1d‐left, the protonation could occur at either the DMA substituent (N(CH_3_)_2_) or the azo moiety (N=N), resulting in either ammonium or azonium ions, respectively.^29^ The dipole moment changes between the neutral and the protonated states are 6.59 and 17.76 D for the azonium and ammonium ions, respectively, and the dipole direction is the same as their neutral states. The ammonium ions generated from protonation can further attract anions to form ion pairs through electrostatic interaction and consequently generate ionic dipoles between the cations and the anions, when there are anions around,^37^, ^38^ as shown by the simulation results in Figure 1d‐right. In addition, the ionic dipole has an opposite dipole direction compared to the neutral case (Figure 1d‐left), and the dipole moment of the ion‐pair shows a strong dependence on the anion size. The dipole moment changes of these ion‐pairs are 6.19, 11.64, and 13.54 D for Cl^−^, Br^−^, and I^−^, respectively, which correlates quite well with that the polarizability of ion‐pairs rises with increasing anion size.^39^, ^40^

The effect of the dipole moment change on SiNR‐FET sensing was examined by measuring the real‐time pH response of the Azo‐modified SiNR‐FETs. The schematic cross‐sectional view of a SiNR‐FET covered by electrolyte for pH sensing is shown in **Figure** **2**a, with a top‐view scanning electron microscope (SEM) image depicting the central part of the device as an inset. A cross‐sectional transmission electron microscope image of the SiNR is shown as Figure S4 in the Supporting Information. The SiNR channel is 2 µm in length, 190 nm in width, and 40 nm in height. An SAM was incorporated on the gate oxide (SiO_2_) surface of the SiNR‐FET. Details of the SiNR‐FET fabrication process^41^ and the monolayer preparation^42^/characterization are presented in Figure S1 and related discussion in the Supporting Information. The SiNR‐FET exhibits a typical p‐type behavior, with a large on to off current ratio (*I*_on_/*I*_off_) exceeding 10^5^ and a low gate leakage current *I*_G_ ≈ 0.5 nA (2.6 × 10^−2^ A cm^−2^) which guarantees a stable device platform for subsequent measurements. The transfer characteristics (*I*_DS_ vs *V*_G_) of a SiNR‐FET modified with different SAMs can be found in Figure S5 (Supporting Information). The measurements were implemented in 1 × 10^−3^ m KCl electrolytes at different pH adjusted with hydrochloric acid and calibrated by commercial pH meter. The working principle of FET‐based ion‐selective pH sensors has been well described in many publications.^43^, ^44^ The inset in Figure 2b shows the pH response of the H‐Azo‐modified SiNR‐FET containing the evolution of drain‐to‐source current (*I*_DS_) with decreasing pH from 5 to 1 and the corresponding surface potential change (Δφ_s_). The responses of other SAM‐modified SiNR‐FETs can be found in Figures S5 and S6 (Supporting Information). To prove the dipole effect, we compared the pH sensitivities of the H‐Azo and CF_3_‐Azo‐modified SiNR‐FETs with that of a reference SiNR‐FET modified with saturated n‐octadecane (OTS) SAM, as shown in Figure 2b. The reference SiNR‐FET shows a constant increase in φ_s_ with decreasing pH, which indicates an increasing amount of protons associated with the surface hydroxyl groups on the gate surface of the SiNR‐FET. However, with decreasing pH, φ_s_ of the H‐Azo‐modified SiNR‐FET increases initially but decreases drastically after pH < 3. This drastic signal transition at pH = 3 matches well with the point when H‐Azo chromophore started to be protonated as shown by the UV spectra (Figure 1b). According to the DFT calculations in Figure 1c, the dipole of the protonated H‐Azo chromophore points toward the substrate. In the following discussions, we denote this dipole direction as negative, while the opposite direction is positive, as shown in Figure 1e. The negative dipole causes an opposite effect to φ_s_ in comparison to that caused by the proton adsorption in hydroxyl groups on the gate surface. This hypothesis was supported by the fact that the CF_3_‐Azo‐functionalized SiNR‐FET behaves similarly to the reference SiNR‐FET with changing pH, as the CF_3_‐Azo chromophore is very difficult to be protonated due the strong electron‐withdrawing power of the CF_3_ substituent.

**Figure 2:**
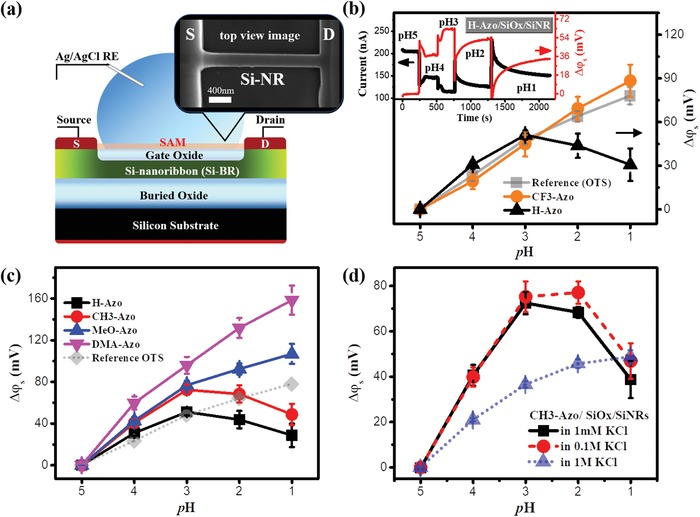
a) Schematic diagram of SiNR‐FET pH sensing setup and SEM image of a SiNR‐FET. b) Δφ_s_ versus pH for SiNR‐FETs modified with CF_3_‐Azo, H‐Azo, and OTS (as the reference) SAMs. Inset: device current (*I*_DS_) and potential change (Δφ_s_) as a function of time at various pH various for the H‐Azo‐modified SiNR‐FET. c) Δφ_s_ versus pH for SiNR‐FETs modified with various SAMs. d) Δφ_s_ versus the pH for the CH_3_‐Azo‐modified SiNR‐FETs in 1 × 10^−3^, 0.1, and 1 m of KCl electrolytes, respectively. The solid dots represent the mean values and their standard deviations are shown as the corresponding error bars.

To further prove the dipole effect on pH response of the SiNR‐FETs, we compared the φ_s_ variation of the SiNR‐FETs modified with CH_3_‐Azo, MeO‐Azo, and DMA‐Azo which contain substituents with relatively stronger electron donation power than that of H‐Azo. According to the DFT calculations in Figure 1c, the dipole moment differences between neutral and protonated are 7.08, 4.79, and 3.46 D for H‐Azo, CH_3_‐Azo, and MeO‐Azo chromophore, respectively. The SiNR‐FET responses followed this trend in dipole moment change very well. In detail, the CH_3_‐Azo‐modified device showed less Δφ_s_ down turn than the H‐Azo device below pH 3. The MeO‐Azo‐modified device had a more linear response to pH with an invisible down turn of Δφ_s_. The DMA‐Azo‐modified showed a much stronger response than the reference device in the whole detection range with no potential down turn. This further confirms that the potential down turn below pH 3 is caused by the molecule dipole changing from positive to negative direction, i.e., a negative change in dipole moment, which has an opposite effect on Δφ_s_ compared to the proton adsorption in hydroxyl groups on the gate surface of the SiNR‐FET. In addition, for the DMA‐Azo‐modified device, it showed a significant potential increase at pH around 5–4 where the DMA‐Azo chromophore was protonated according to the UV studies in Figure 1b. This was consistent with the large enhancement of the positive dipole contributed by the Cl^−^ as shown in Figure 1d‐right. In addition, we notice that the CF_3_‐Azo‐ and H‐Azo‐modified devices have similar potential responses to that of the OTS‐modified device above pH 3, where their azo chromophores have not been protonated. It indicates that their Δφ_s_ at pH > 3 originates from the same contribution from the proton interactions with the surface hydroxyl group.^45^ But we also notice that Δφ_s_ of the CH_3_‐Azo‐ and the MeO‐Azo‐modified devices are higher than that of the H‐Azo‐modified device in the pH > 3 region. It was most likely due to the increased basicity of the azo chromophore caused by stronger electron‐donating substituents (CH_3_ and MeO groups), leading to the accumulation of protons via the acid–base electrostatic interactions near the surface molecule accordingly. This physical interaction is similar to that between protons and hydroxyl groups.

Furthermore, the synergy of ionic and dipolar effects on the chromophore‐functionalized SiNR‐FET was examined at different ionic strengths of the electrolyte. The ionic strength indeed could affect the sensing signal by influencing the Debye length, since only the charge or molecular dipole change within the Debye length could contribute to the sensing signal. This is evidenced by the pH responses of the CH_3_‐Azo‐modified devices in KCl electrolytes with the ionic strengths of 1 × 10^−3^, 0.1, and 1 m (Debye length 10, 1, and 0.3 nm, respectively), as shown in Figure 2d and Figure S7 (Supporting Information). When the ionic strength increases from 1 × 10^−3^ to 0.1 m, the Δφ_s_ down turn below pH 3 is reduced, indicating that the dipole moment change in the molecule is partially screened. Further increase of the ionic strength to 1 m almost eliminates the Δφ_s_ down turn below pH 3 and also largely suppresses the pH response above pH 3, implying that both the dipole moment change and the proton accumulation via the acid–base electrostatic interactions in the molecules are screened when the Debye length is reduced to 0.3 nm. Hence, high salt concentration need to be avoided in sensing applications in order to benefit from the synergy of ionic and dipolar effects by molecular design. **Figure** **3**a summaries how Δφ_s_ can be affected by changes in both charge density and dipole moment on the SiNR‐FETs modified with different azo SAMs. There are two types of physical interactions that can accumulate protons on the device surface and contribute to Δφ_s_. One is the proton‐hydroxyl group interaction as illustrated as C_1_ in Figure 3a. The other one is the acid–basic electrostatic interaction between proton and the azo chromophore dependent upon the electron‐donating power of substituents (noted as C_2_ in Figure 3a). Besides, when the proton concentration is high enough, they can chemically react with the surface molecules, generate changes in dipole moments and contribute to Δφ_s_. Such a chemical reaction between proton and the azo moiety is illustrated as D_1_ in Figure 3a. This type of reaction will generate the molecular dipole with a negative direction (toward the device surface) as a result from the protonated azo, as shown in Figure 1c,d‐left. The dipole moment change in this direction will in turn reduce Δφ_s_. However, when the basicity of the substituent is very strong, as the case of DMA‐Azo, the protons can react directly with the substituents, illustrated as D_2_ in Figure 3a. The positively charged protonated substituent, ammonium, will attract an anion in the electrolyte, forming an ion pair with a strong ionic dipole accordingly. This ionic dipole of the DMA‐Azo chromophore is in positive direction (toward the bulk electrolyte) according to the DFT calculations shown in Figure 1d‐right, which will increase Δφ_s_. An analytical model based on the equivalent circuit shown at the right of Figure 3a was constructed to calculate Δφ_s_ considering both charge and dipole effects on the sensing surface (detailed model description is available in the Supporting Information). The measured Δφ_s_ could be well fitted with the model as shown in Figure 3b. To further illustrate the effects of the surface charge and dipole on Δφ_s_, a 3D mapping reflecting the dependence of Δφ_s_ on dipole moment and solution pH simulated using the model is shown in Figure 3c. It is evident from the simulated results that positive changes in dipole moment will increase Δφ_s_ of the SiNR‐FET, while negative changes in dipole moment will suppress Δφ_s_, in comparison to Δφ_s_ for the zero‐dipole case (i.e., the molecular dipole is invariant with pH), as marked by the red line on the surface in Figure 3c. The larger the dipole moment, the more obvious the effect is expected.

**Figure 3:**
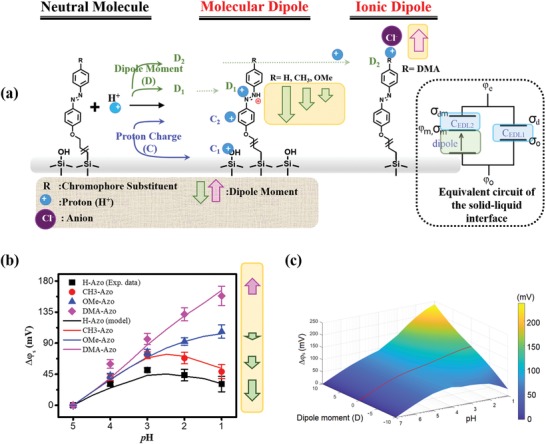
a) Schematics showing the mechanism of protonation and adsorption processes of the azo‐chromophore‐functionalized molecules and the hydroxyl groups of SiO_2_ on the device surface. Herein, D_1_ and D_2_ represent the dipole effects and corresponding protonation sites (D_1_: the azo moiety of chromophore and D_2_: the chromophore substituent); C_1_ and C_2_ indicate the charge effects and corresponding active sites (C_1_: the hydroxyl group of SiO_2_ and C_2_: the azo chromophore. Right: equivalent circuit of the solid–liquid interface, in which φ_o_ and φ_e_ are the potential of the sensor surface and electrolyte, respectively. φ_m_ is the potential at the functionalized molecular monolayer. σ_o_ and σ_m_ are the fixed charge density on the oxide surface and the functionalized molecular monolayer, while σ_d_ and σ_md_ represent the charge density at the diffuse layer in the electrolyte caused by σ_o_ and σ_m_, respectively. b) The potential change (Δφ_s_) versus pH for the devices studied. The solid points are experimental data showing the mean values and their standard deviation are displayed as the error bars. The lines are the fitting results by our analytical model. c) Δφ_s_ as a function of the dipole moment change associated with pH predicted by our model. The surface functionalized molecule density adopted in this calculation is 3 × 10^18^ m^−2^, and the hydroxy group density is 5 × 10^17^ m^−2^. The dipole moment change with pH follows the UV spectrum of H‐Azo. The red line marks the zero‐dipole situation that the molecular dipole is invariant with pH.

As pointed out in the model, Δφ_s_ can be increased by the positive dipole moment change. Meanwhile, the positive ionic dipole moment formed in the DMA‐Azo chromophore can be modulated by the anion size. To verify this, the pH response of the DMA‐Azo‐modified devices was measured in 1 × 10^−3^ m potassium halide electrolytes, KCl, KBr, and KI with different anion sizes in the order: Cl^−^ < Br^−^ < I^−^. The measured results are shown in **Figure** **4**a and Figure S8 (Supporting Information). Since the DMA‐Azo chromophore was protonated at around pH 5–4 according to the UV studies, the pH response was measured in the pH range from 6 to 1. As predicted, the potential response exhibits a clear dependence on the anion size in the pH range from 5 to 3, where the protonation reaction happens. The device sensitivity increased from 48.0 mV pH^−1^ with Cl^−^ to 60.0 mV pH^−1^ with I^−^ in this pH range. To further prove that the positive ionic dipole results from the protonation of the amine substituent in the DMA‐Azo chromophore, as well as its effect on the potential response, molecules with only amine groups (without the azo chromophore) were used to functionalize the SiNR‐FETs. Δφ_s_ as a function of pH for SiNR‐FETs functionalized with SAMs of the monoamine, diamine, and triamine molecules is summarized in Figure 4b and Figure S9 (Supporting Information). Molecular structures and corresponding abbreviations of these amine‐carrying molecules are shown in the inset of Figure 4b. All the amine‐modified SiNR‐FETs showed a strong enhancement of pH sensitivity comparing to that of the reference OTS‐modified SiNR‐FET for pH 5–2. Moreover, the sensitivity enhancement is also dependent on the number of amines in the molecule (triamine > diamine > monoamine molecules). These results confirm that the amine groups indeed play a key role in the formation of positive ionic‐dipoles, and the pH sensitivity can be boosted by introducing more amines in the molecule. It is worth noting that the average sensitivity in the pH 5–3 was 57.8, 74.6, and 87.6 mV pH^−1^ for the monoamine‐, the diamine‐, and the triamine‐modified SiNR‐FETs, respectively. Benefiting from the contribution of the dipole moment changes, the best sensitivity obtained on the amine‐modified SiNR‐FET is more than four times higher than that of the reference OTS‐modified SiNR‐FET (19.6 mV pH^−1^), with an over two times increase than that of the bare SiO_2_‐based SiNR‐FET (23–36 mV pH^−1^) and is above the Nernstian limit. Figure 4c summarizes the sensing performance of our devices in comparison with reported pH sensors in the literature.^46^, ^47^, ^48^, ^49^, ^50^, ^51^, ^52^, ^53^, ^54^, ^55^, ^56^ The demonstrated potentiometric pH sensing signal using our novel interface engineering is clearly more advantageous.

**Figure 4:**
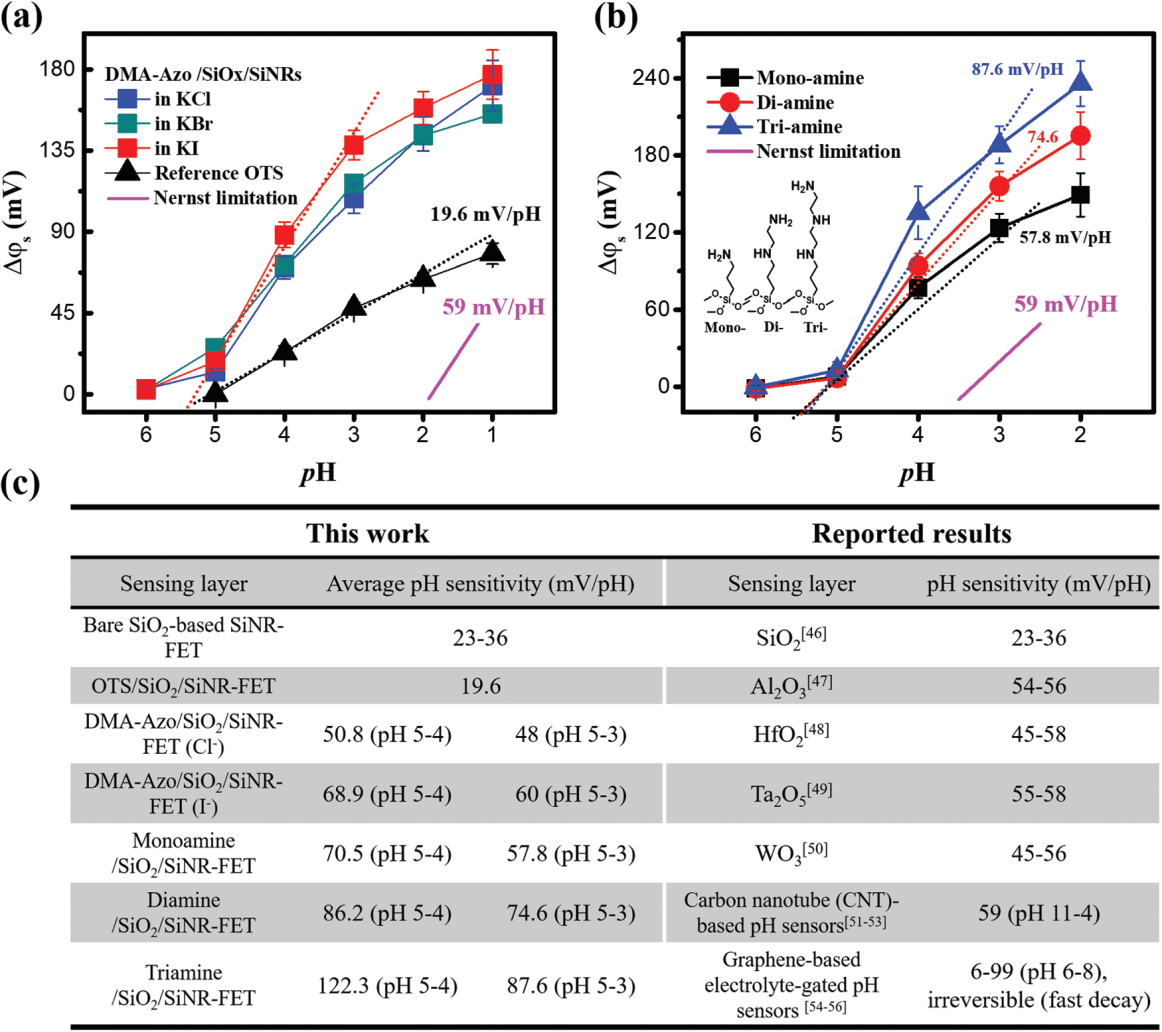
a) Δφ_s_ versus pH for the DMA‐Azo‐modified devices measured in 1 × 10^−3^ m solutions of different potassium halide electrolytes, KCl, KBr, and KI. The result from the OTS‐modified device measured in 1 × 10^−3^ m KCl is included for comparison. b) Δφ_s_ versus pH for SiNR‐FETs modified with monoamine‐, diamine‐, and triamine‐SAMs measured in 1 × 10^−3^ m KCl. Inset: the molecular structures and abbreviations of the amine‐carrying molecules. c) The comparison of the performance of our pH‐sensors and other reported potentiometric devices in literature.

This work reports a strategy to enhance the sensitivity in potentiometric sensing by functionalizing the sensor surface with a chromophore monolayer. With proper chromophore molecule design, the interactions between protons and the chromophore monolayer can generate strong changes in surface dipoles, combined with the surface charge effect, resulting in significantly enhancing pH sensitivity. Especially, the dipole effect plays a critical role in the sensing signal. We demonstrated that the dipole effect renders systematical controllability in both magnitude and direction, which can be achieved not only by controlling of the chromophore polarizability but also the chromophore‐anion interaction after protonation of the chromophore. Benefiting from the synergy effect of dipole and charge on the surface, a super Nernstian pH sensitivity of 87.6 mV dec^−1^ in the pH range 5–3 can be achieved, which is very promising in biosensor applications. Our approach offers the possibility to understand and tune the interaction between analytes and sensor surface. Future systematic studies on aspects of the ionic dipole effect (size, shape, and related counterion position of ion‐pairs), and the mechanism (the analyte–molecule interaction and the ion–molecule interaction) are required to allow further optimization of the associated effects and facilitate design of more comprehensive molecules to reach better sensitivity over larger pH ranges. Furthermore, extending the knowledge to other sensing systems will diversify the receptor design and enable both charge and dipole effects to modulate and enhance sensing sensitivity for a wide scope of applications.

## Conflict of Interest

The authors declare no conflict of interest.

## Author Contribution

Z.Z. initiated, supervised the project, and revised the paper. C.‐W.T. conceived, designed, and performed the sensing experiments and analyzed the data. D.‐C.H. synthesized and characterized all azobenezene compounds. C.W. constructed the theoretical model and discussed the results. C.‐H.L. did the density functional theoretical calculations. S.C., Q.H., and X.C. fabricated the Si‐NR chip. X.X. assisted with the sample preparation. S.‐L.Z. and Y.‐T.T. discussed and commented the experiments. The manuscript was written by C.‐W.T. All the co‐authors reviewed and agreed with the final version of the manuscript.

## Supporting information

Supporting Information
