# Supplementary material for: Synergy of Ionic and Dipolar Effects by Molecular Design for pH Sensing beyond the Nernstian Limit
Source: Adv Sci (Weinh). 2019 Nov 27;7(2):1901001. doi: 10.1002/advs.201901001 (PMC6974946; doi:10.1002/advs.201901001)
Supplement: Supplementary file 1 — Supporting Information [file ADVS-7-1901001-s001.pdf]

Copyright WILEY-VCH Verlag GmbH & Co. KGaA, 69469 Weinheim, Germany, 2019.

ADVANCED SCIENCE
Open Access

Supporting Information

for *Adv. Sci.,* DOI: 10.1002/advs.201901001

Synergy of Ionic and Dipolar Effects by Molecular Design for pH Sensing beyond the Nernstian Limit

*Chiao-Wei Tseng, Chenyu Wen, Ding-Chi Huang, Chin-Hung Lai, Si Chen, Qitao Hu, Xi Chen, Xingxing Xu, Shi-Li Zhang, Yu-Tai Tao, and Zhen Zhang**

Copyright WILEY-VCH Verlag GmbH & Co. KGaA, 69469 Weinheim, Germany, 2019.

Supporting Information

Synergy of ionic and dipolar effects by molecular design for pH sensing beyond the Nernstian limit

*Chiao-Wei Tseng, Chenyu Wen, Ding-Chi Huang, Chin-Hung Lai, Si Chen, Qitao Hu, Xi Chen, Xingxing Xu, Shi-Li Zhang, Yu-Tai Tao, and Zhen Zhang**

Dr. C.-W. Tseng, C. Wen, Dr. S. Chen, Q. Hu, X. Chen, X. Xu, Prof. S.-L. Zhang, Prof. Z. Zhang
Division of Solid-State Electronics, The Ångström Laboratory, Uppsala University, SE-751 21 Uppsala, Sweden
E-mail: zhen.zhang@angstrom.uu.se
Dr. D.-C. Huang, Prof. Y.-T. Tao
Institute of Chemistry, Academia Sinica, Taipei, Taiwan
Prof. C.-H. Lai
Department of Medical Applied Chemistry, Chung Shan Medical University, Taichung 40201, Taiwan

Table of Contents:

1. Experimental section
2. Computational dipole moment
3. Tables and Figures
Table S1. The nucleophilicity of the active atoms of the chromophore as well as the Gibbs free energy (ΔG) of the protonated species for all the studied chromophores.
Figure S1. Characterization of the azo chromophore and the OTS monolayers.
Figure S2. Characterization of the amine-functionalized monolayers.
Figure S3. The UV-vis absorption spectra of the azo chromophores.
Figure S4. A cross-sectional TEM image of our SiNR-FET device.
Figure S5. The transfer characteristics of the SiNR-FET devices with various SAM modifications: (a) CF_3-Azo, (b) H-Azo, (c) CH_3-Azo, (d) MeO-Azo and (e) DMA-Azo.
Figure S6. The device current (I_{DS}) and the potential change ($\Delta\varphi$) as a function of time under various pH for the SiNR-FET with various SAM modifications: (a) CF_3-Azo, (b) CH_3-Azo, (c) MeO-Azo and (d) DMA-Azo.
Figure S7. The device current (I_{DS}) and the potential change ($\Delta\varphi$) as a function of time under various pH for the SiNR-FET modified with CH_3-Azo in KCl electrolytes with the different ionic strength: (a) 1 mM, (b) 0.1 M and (c) 1 M.
Figure S8. The transfer characteristics and the real-time pH response (I_{DS} and $\Delta\varphi$) for the DMA-Azo-modified SiNR-FET devices in the 1mM of (a, b) KCl, (c, d) KBr and (e, f) KI solutions, respectively.
Figure S9. The transfer characteristics and the real-time pH response (I_{DS} and $\Delta\varphi$) for the various amine-modified SiNR-FET devices: (a, b) monoamine-modified devices, (c, d) diamine-modified devices and (e, f) triamine-modifies devices in 1mM KCl electrolyte, respectively.
4. Modelling the pH sensing with functionalized surface containing dipole
5. Synthetic procedures and characterization
6. Reference

1. Experimental section
Sample preparation:
Self-assembled monolayer (SAM):
n-Octadecyltrimethoxysilane was obtained commercially. All azobenzene-containing trimethoxysilanes were synthesized in the laboratory and fully characterized by ^{1}H NMR and ^{13}C NMR, mass spectrometer (details shown in the section of **Synthetic procedures and characterization**). Self-assembled monolayers (SAMs) were prepared by immersing freshly cleaned SiO_2/Si substrates (including the SiNRs-FET chips and the references of planar SiO_2/Si substrates) with oxygen plasma in a 1 mM tetrahydrofuran (THF) solution for 4 hr., followed by thorough rinse with pure THF. Adsorption of the silanes on a planar SiO_2/Si substrate was carried out in parallel as the control samples for monolayer formation and characterization. The above condition for monolayer preparation was further optimized based on our previous studies.[1] In this work, the water contact angle and ellipsometry measurements were used to monitor the formation process as well as confirm the presence of a monolayer. After self-assembly of the monolayers, nearly a constant value of contact angle and a limiting film thickness were obtained in all cases (**Figure S1**a, b). Moreover, for the amine-functionalized monolayer, 3-aminopropyltrimethoxylsilane (APS), N-[3-(Trimethoxylsilyl)propyl]-ethylenediamine (DA) and N-[3-(Trimethoxylsilyl)propyl]diethylenetriamine (TA) were used for preparation of the monoamine, the diamine and the triamine monolayers, respectively. These monolayers were prepared by immersing the device chips and the references substrates in 1% (v/v) APS, DA and TA solutions using a mixture of acetone and water (5:1, v/v) as the solvent for 1 hr., following by sonicating in acetone for 5 min and thorough rinse with acetone, respectively.[2,3] The prepared monoamine, diamine and triamine films showed the average thicknesses of 0.66, 0.92 and 1.58 nm, respectively (**Figure S2**a). These values are very close to the theoretical molecular length of APS (0.6 nm), DS (1.0 nm) and TA (1.5 nm), respectively. Water contact angle values for these films were also measure as 44º, 40º and 39º for the monoamine, diamine and triamine samples, respectively, as shown in **Figure S2a**.

Silicon-nanoribbon transistor (SiNR-FET)
The SiNR-FET chips were manufactured according to the previous process.[4,5] Starting from a silicon-on-insulator (SOI) wafer with the buried oxide thickness (BOX) of 150 nm and the lightly doped ($10^{15}/cm^3$) p-type (100) top silicon thickness of 260 nm. The top silicon layer was first thinned down to 40 nm by thermal oxidation. Then the SiNR channels and the source/drain contact pads were defined using lithography followed by masked etching process. The source/drain pads were doped with heavy p-type dopants via implantation and contacted by a 40 nm thick platinum silicide layer afterwards. Finally, a thin silicon oxide layer was deposited as the gate insulator and the passivation oxide on the electrode pads.[6]

Measurement:
The electrical characteristics of the transistor devices were carried out using Ag/AgCl as a gate electrode in the supporting electrolyte of potassium chloride (KCl) solution under ambient conditions, which were recorded by a keysight B1500A Parameter Analyzer. The pH measurements were implemented in 1 mM KCl electrolytes at different pH adjusted by hydrochloric acid and calibrated by commercial pH meter. The transfer characteristic was obtained at a constant source-drain voltage of 1 V with applied gate bias from 0.5 to -0.5 V. In the real-time drain current measurement, both gate bias (V_G) and drain bias (V_{DS}) kept constant, the current was recorded until it tends to stabilize at the corresponding pH. Among which, the applied gate bias near around its subthreshold voltage was determined by transfer characteristic of the device. The solution pH was changed with hydrochloric acid solution from 5 to 1, while the concentration of KCl electrolyte was kept constant (1mM) during the

measurement. However, in the DMA-Azo device, in order to clarify the device response at the pH above 3, the pH was changed from 6 to 1. Herein, the surface potential (Ψ_s) was extracted from I_{sd} - V_{ref} sweeps and the relation presents by the transconductance ($g_m = \partial I_{sd}/\partial V_{ref}$) according to $\Delta\ \Psi_s = -\Delta V_{th} = -(\Delta I)/g_m$.[7] All surface potential shifts are retrieved with respect to the potential at the pH 5 or 6, so that $\Delta\Psi_s = 0$ at pH 5 or 6. Four devices were measured and their average and standard errors of the mean are plotted. Moreover, for studies of the anion effect, the measurement was carried out in the 1mM potassium halide solutions of KCl, KBr and KI, respectively. Otherwise, the electronic absorption spectroscopy was detected by using a Jasco V-530 double beam spectrophotometer. The UV measurement was carried out in dichloromethane with the solution pH (changing from 5 to 0) adjusted with trifluoroacetic acid due to the azo chromophores with poor solubility in water. The concentration of chromophore solution was 10^{-5} M. In addition, the contact angle was measured by the DIGIDROP GBX contact angle meter using ultrapure water as the wetting liquid, and besides, the thickness of the monolayer was measured by an ellipsometer.

2. Computational dipole moment

In the context of this study, all the stationary points were modeled in terms of quantum-mechanical calculations. The DFT method employing the B3LYP functional was corrected by the D2 version of Grimme's dispersion.[8,9] In conjunction with the def 2-SVP basis set,[10] it was used to carry out the full optimization of the compounds of interest in the gas phase. After the geometry optimization was converged, the vibrational frequency analysis at the same theoretical level was performed to confirm that the number of imaginary frequencies for the stationary point was zero. Both the geometry optimizations and vibrational frequency analyses were done with the Gaussian 09 package.[11] **Table S1** shows the nucleophilicity of the atoms (N_α, N_β and N_γ) as well as the Gibbs free energy (ΔG) of the corresponding protonated species for all azo chromophore molecules studied. The abbreviations of N_α, N_β and N_γ representing the nitrogen atoms of the azo moiety and the active atom (such as O and N) of the substituent are shown in the structure image at the left side of the table. The result was used to help determine the protonated site (atom) of the chromophore. For the OMe-substituted chromophore (OMe-Azo), although the oxygen atom of the OMe group has a stronger nucleophilicity in the structure, the large ΔG could make the substituent less favorable for protonation. Therefore, it was supposed that the protonation preferably occurs at the azo moiety rather than the OMe group. Besides, due to two substituents of the azo moiety with very similar properties, the nucleophilicity of the two nitrogen atoms is very close, and the polarizability of chromophore is poor for the OMe-Azo chromophore. Moreover, in the case of DMA-substituted chromophore (DMA-Azo), the nitrogen of the DMA substituent has much stronger nucleophilicity than that of the azo moiety, but the generated azonium ion are more stable than the ammonium ion after protonation. Furthermore, compared with the OMe-Azo case, the ΔG is significantly smaller in the case of DMA-Azo.

3. Table and Figure

Table S1. The nucleophilicity of the active atoms (N_α, N_β and N_γ) of the chromophore as well as the Gibbs free energy (ΔG) of the protonated species for all the studied chromophores. The inset shows the chemical structure of the azo chromophore. N_α and N_β represent the nitrogen atoms of the azo moiety as well as the N_γ means the active atom of the chromophore substituent.

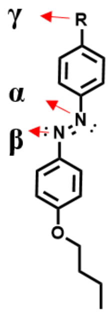

			CF3-Azo	H-Azo	CH3-Azo	OMe-Azo	DMA-Azo
Neutral State	Nucleophilicity	Nα	-0.1850	-0.1746	-0.1634	-0.1494	-0.1443
		Nβ	-0.0891	-0.1201	-0.1347	-0.1462	-0.1556
		Nγ	–	–	–	-0.3671	-0.2593
Protonated State	ΔG (ΔE) In kcal/mol	Nα	0.0	0.0	0.0	0.0	0.0
		Nβ	5.3 (5.4)	4.1 (4.0)	2.6 (2.6)	0.4 (0.4)	-4.3 (-3.1)
		Nγ	–	–	–	31.7 (32.6)	2.5 (3.1)

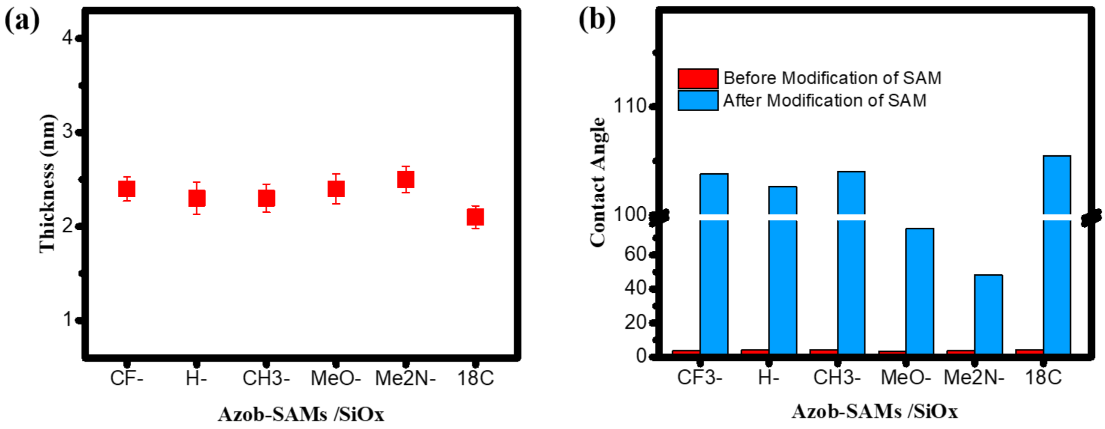

Figure S1. Characterization of the azo chromophore and the OTS monolayers: (a) Monolayer thickness measured by the ellipsometry. (b) Substrate water contact angle before and after modification of monolayers.

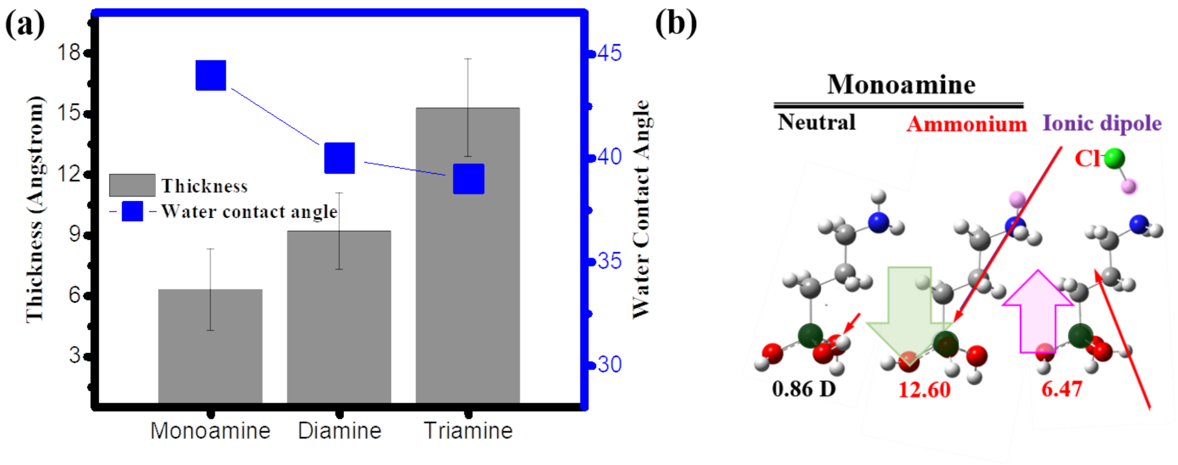

Figure S2. Characterization of the amine-functionalized monolayers: (a) Ellipsometric thicknesses and water contact angles of the monoamine, diamine and triamine films. (b) The left two: The DFT calculations of dipole moment for the monoamine (APS) molecule at neutral and protonated states respectively. Right: The DFT calculations of dipole moment for the ion-pair between the protonated molecule and the anion Cl^-. (The colored balls on the structure represent the atom: black (carbon), grey (hydrogen), red (oxygen), dark green (silicon), blue (nitrogen) and pink (the hydrogen from protonation). The arrows indicate the dipole moment: red (the DFT calculation showing the amplitude and direction of dipoles and the corresponding value are listed at the bottom of structures), green and pink (the dipole moment change for each case, comparing to the corresponding neutral case).)

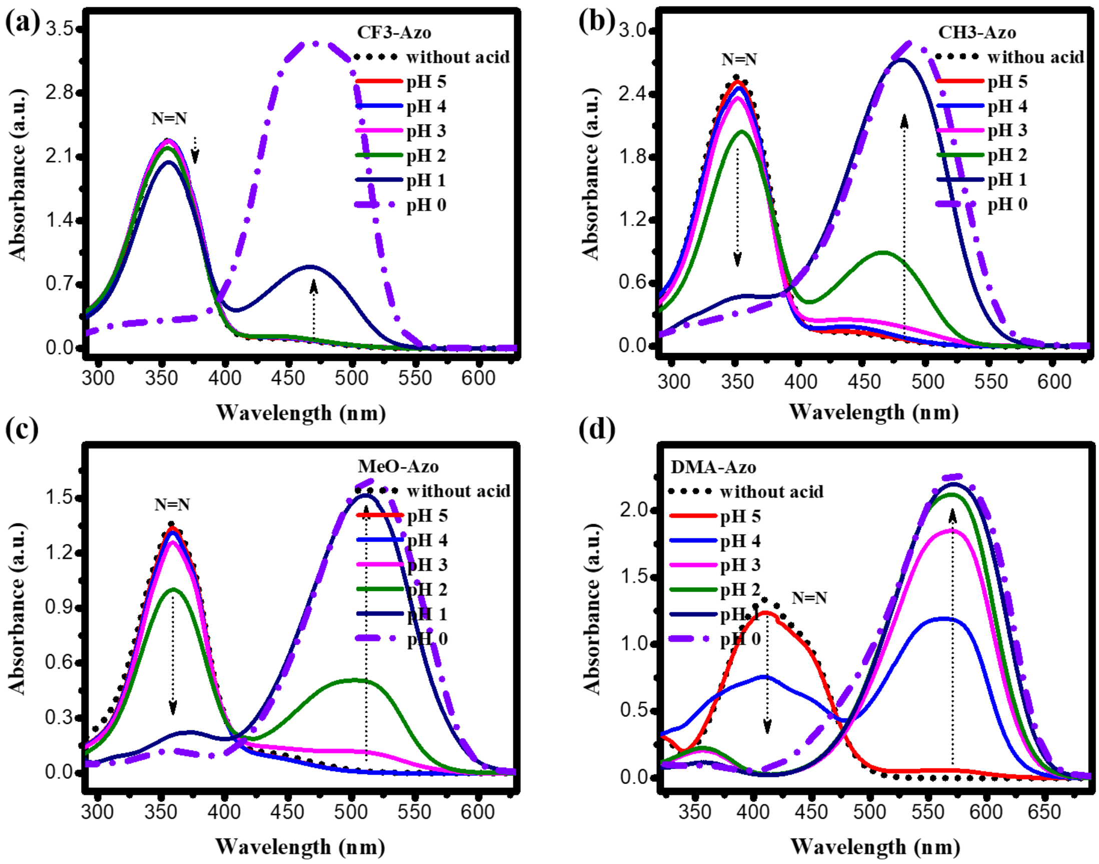

Figure S3. The UV-vis absorption spectra of the azo chromophores: (a) CF_3-Azo, (b) CH_3-Azo, (c) MeO-Azo and (d) DMA-Azo at various solution pH, respectively. The UV measurement was carried out in dichloromethane with the solution pH changing from 5 to 0 due to the azo chromophores with poor solubility in water.

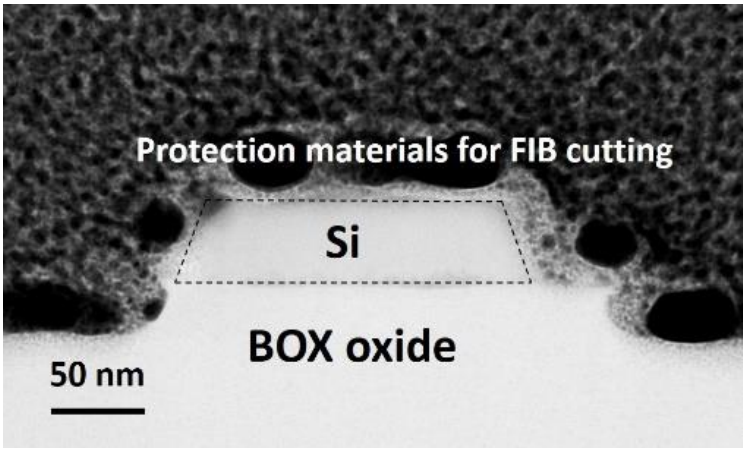

Figure S4: A cross-sectional TEM image of a SiNR-FET device.

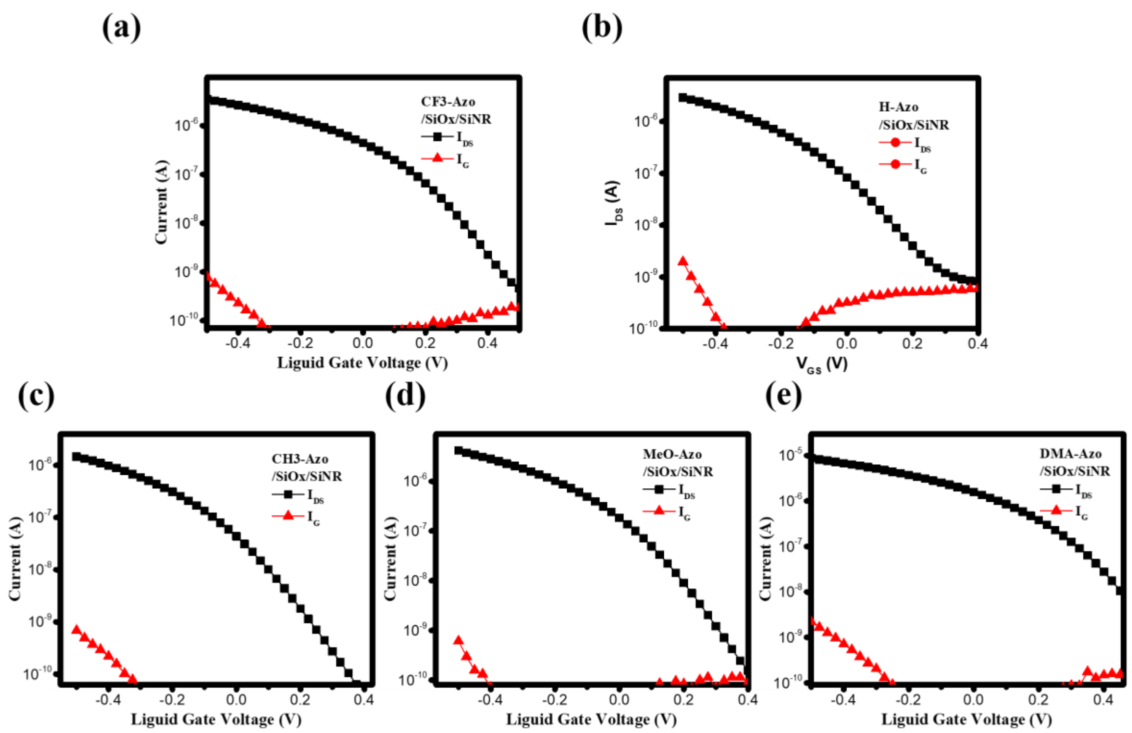

Figure S5. The transfer characteristics of the SiNR-FET devices with various SAM modifications (1 mM KCl, pH 5): (a) CF_3-Azo, (b) H-Azo, (c) CH_3-Azo, (d) MeO-Azo and (e) DMA-Azo.

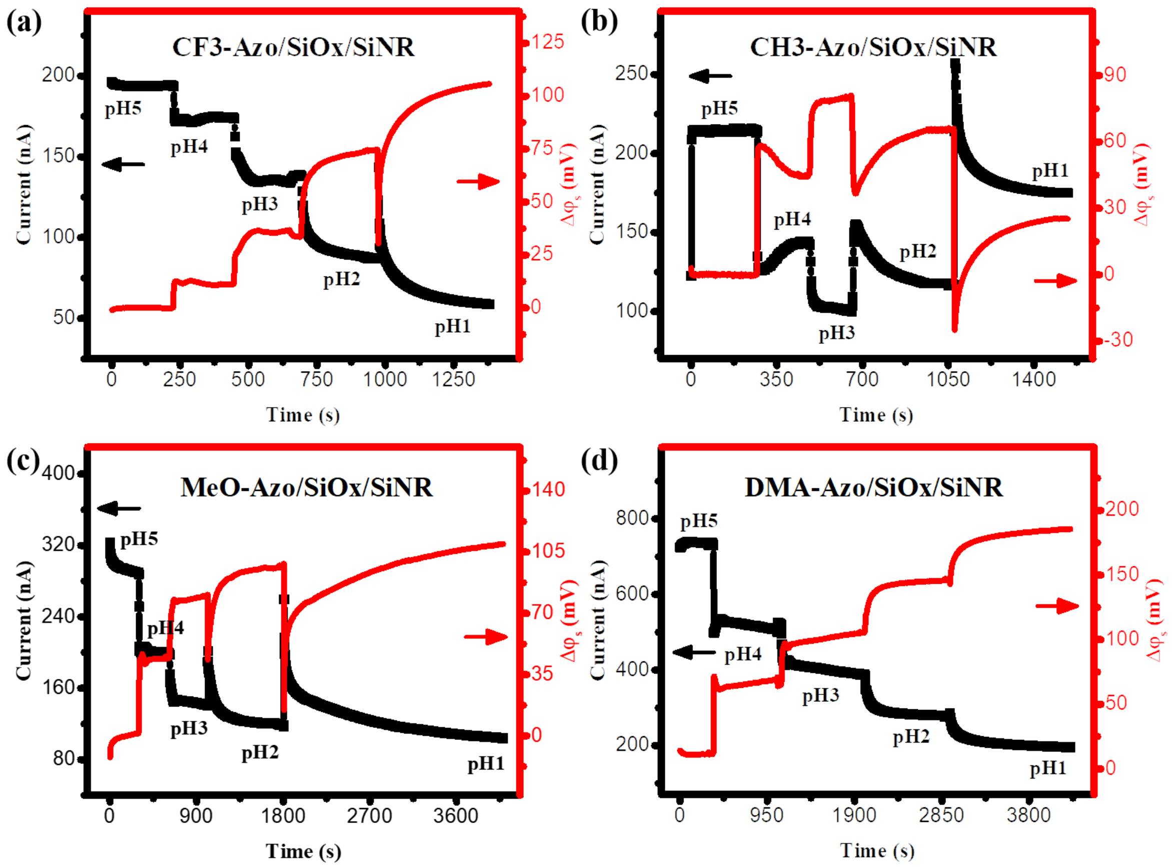

Figure S6. The device current (I_{DS}) and the potential change ($\Delta\varphi$) as a function of time under various pH for the SiNR-FET with various SAM modifications: (a) CF_3-Azo, (b) CH_3-Azo, (c) MeO-Azo and (d) DMA-Azo.

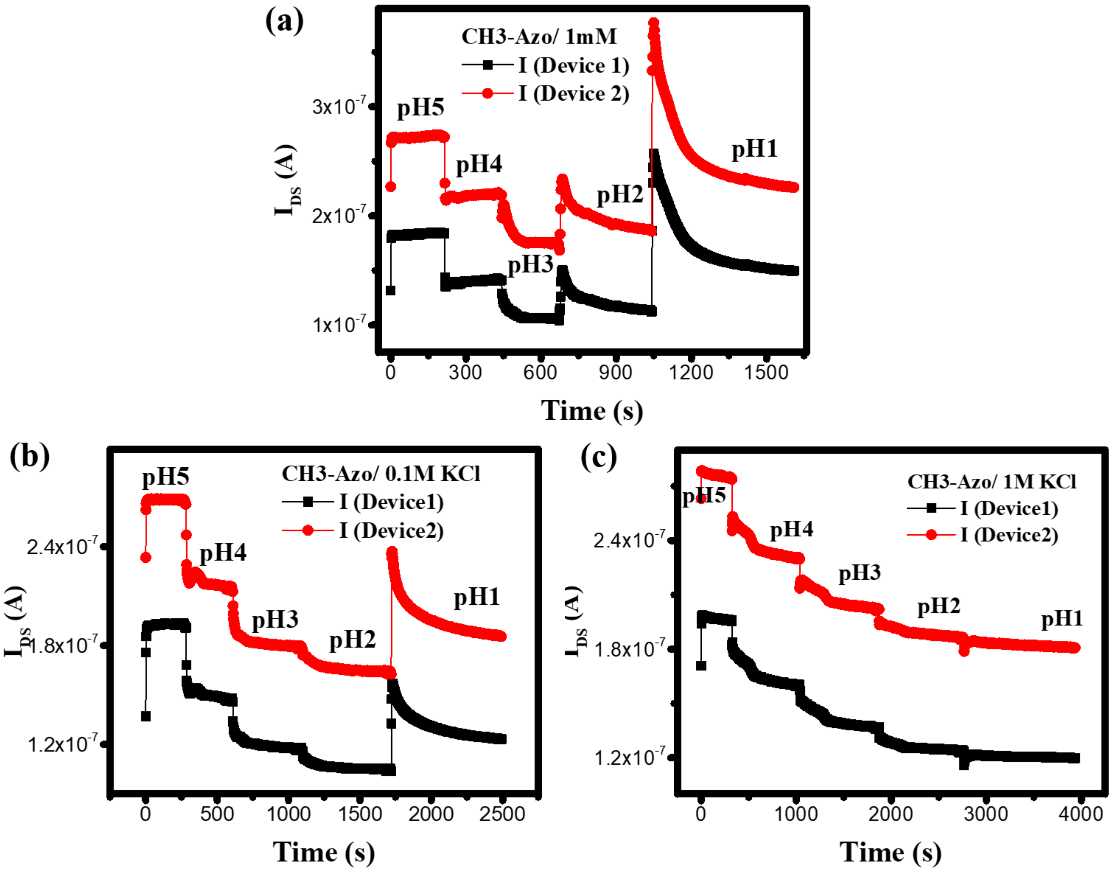

Figure S7. The channel current (I_{DS}) and the potential change ($\Delta\varphi$) as a function of time under various pH for the SiNR-FET modified with CH_3-Azo in KCl electrolytes with the different ionic strength: (a) 1 mM, (b) 0.1 M, and (c) 1 M.

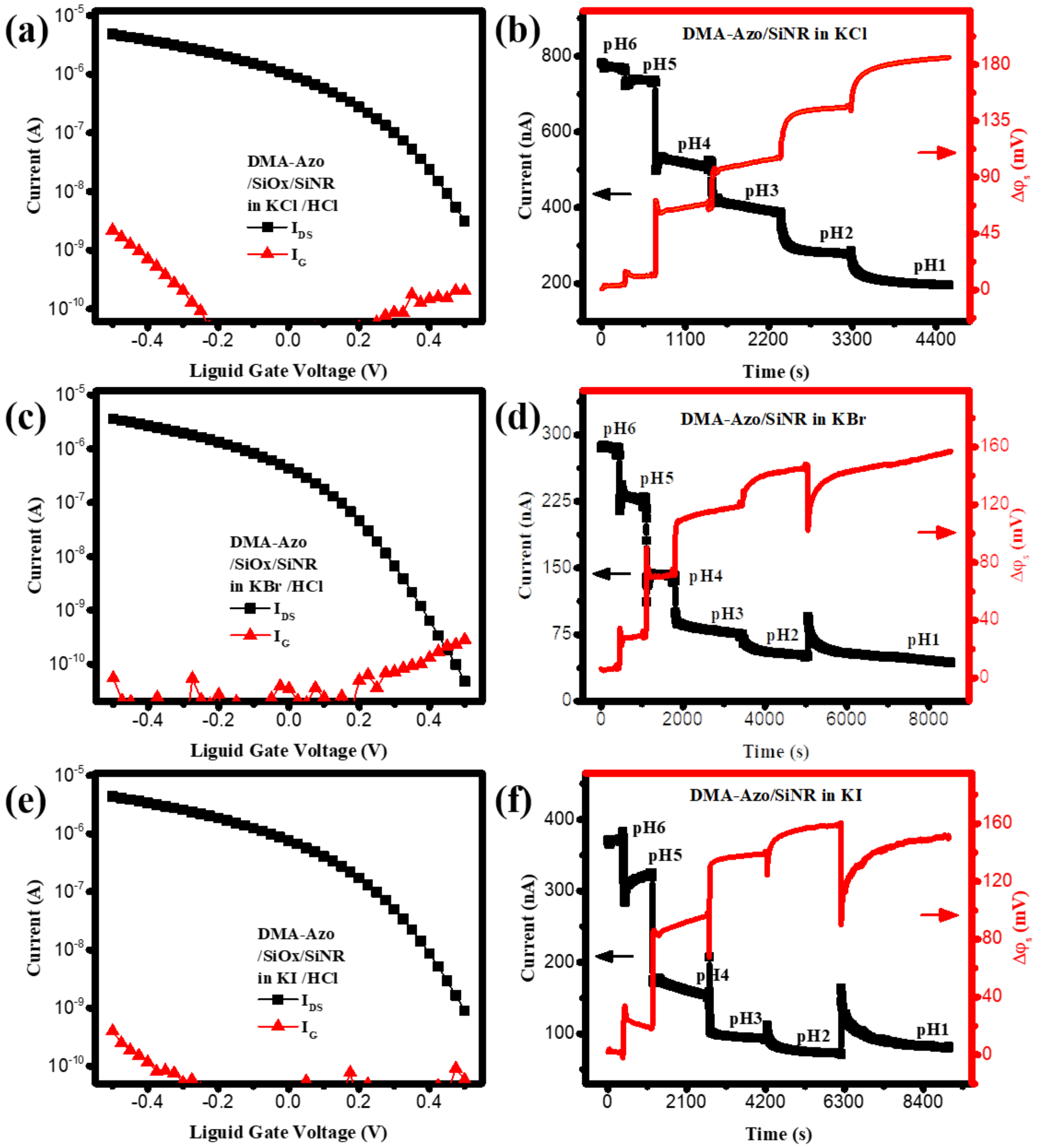

Figure S8. The transfer characteristics (1 mM KCl, pH 6) and the real-time pH response (I_{DS} and $\Delta\varphi$) for the DMA-Azo-modified SiNR-FET devices in the 1mM of (a, b) KCl, (c, d) KBr and (e, f) KI solutions, respectively.

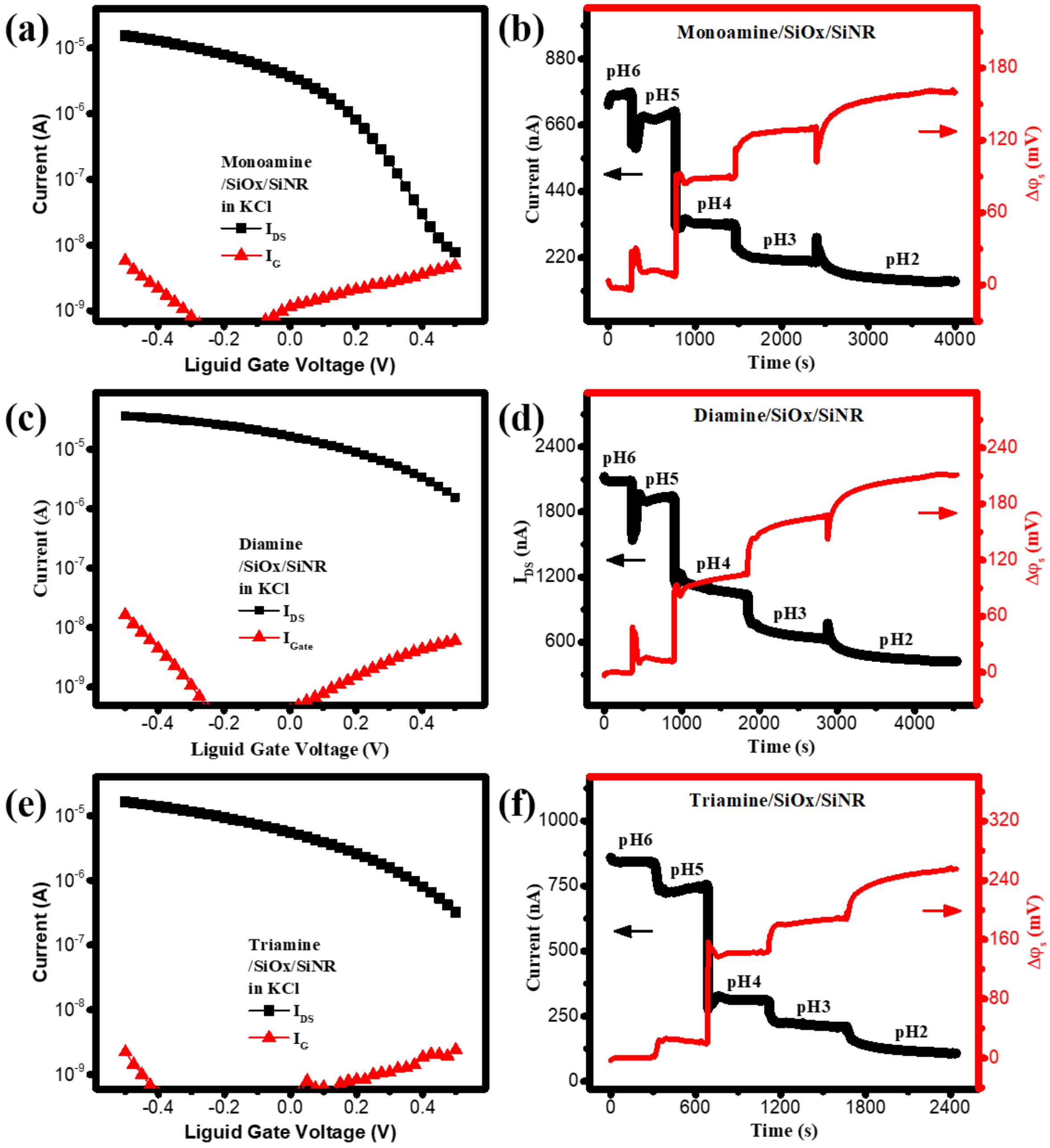

Figure S9. The transfer characteristics (1 mM KCl, pH 6) and the real-time pH response (I_{DS} and $\Delta\varphi$) for the various amine-modified SiNR-FET devices: (a, b) monoamine-modified devices, (c, d) diamine-modified devices and (e, f) triamine-modifies devices in 1mM KCl electrolyte, respectively.

4. Modelling the pH sensing with functionalized surface containing dipole

Model description:

Based on site-binding model, the adsorption sites of on solid surface tend to reach equilibrium state of adsorption-desorption with hydrogen ions in electrolyte. However, in our system, there are two kinds of sites on the surface. One is the hydroxyl group from the uncovered area of SiO_2, as shown in **Figure S10**(a). These groups interact with hydrogen ions and reach equilibrium state following the processes below:

$$-Si-OH+H_2O \xleftrightarrow{K_a} -Si-O^- + H_3O^+$$

$$-Si-OH+H_3O^+ \xleftrightarrow{K_b} -Si-OH+H_2O$$

The interface behaves as a capacitor forming the electric double layer (as shown the right branch in **Figure S10**(b)). According to the site binding model, the equilibrium is described by two equations:[12,13]

$$\sigma_0 = qN_s \frac{[H^+]^2 exp(\frac{2q\varphi_{eo}}{kT}) - K_aK_b}{K_aK_b + K_a[H^+]exp(\frac{q\varphi_{eo}}{kT}) + [H^+]^2 exp(\frac{2q\varphi_{eo}}{kT})} \tag{S-1}$$

$$\varphi_{eo} = \frac{2kT}{q} arcsinh(\frac{\sigma_d}{\sqrt{8\varepsilon_0\varepsilon_w kTN_A c}}) + \frac{\sigma_d}{C_{stern}} \tag{S-2}$$

Where, $[H^+]$ is the concentration of hydrogen ions in the bulk of electrolyte, q is the unit charge, N_s is active site density, k is the Boltzmann constant, T is temperature in Kelvin, K_a and K_b are the equilibrium constants of the aforementioned reactions, ε_0 is the vacuum dielectric constant, ε_w is relative dielectric constant of water, N_A is the Avogadro constant, c is the ion concentration of electrolyte, and C_{stern} is the stern capacitance in unit area. All the charge density and potential at different position in equivalent circuit, denoted as σ and φ with corresponding subscripts respectively, are marked in **Figure S10** (b).

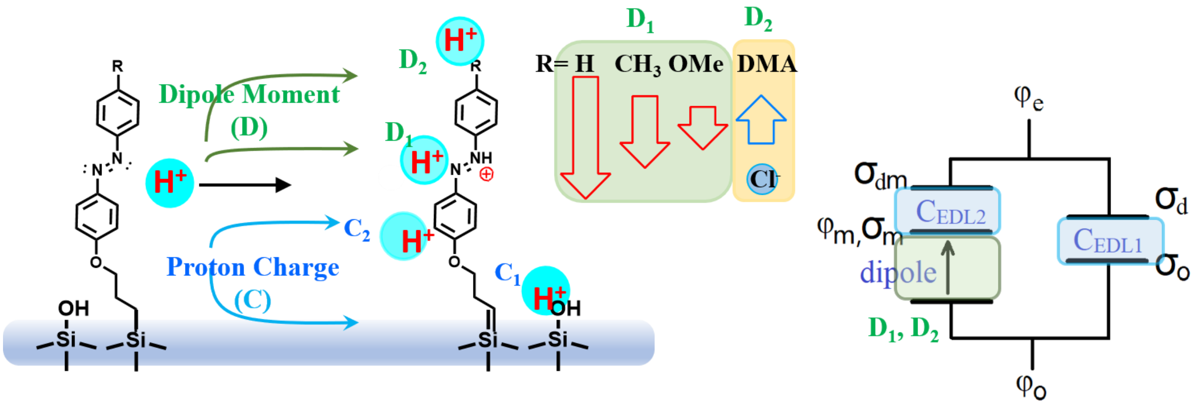

Figure S10 (a) Schematics of the mechanism of protonation-deprotonation processes of functionalized molecules and uncovered SiO_2 sites on the device surface. (b) Equivalent circuit of the solid-liquid interface.

The other kind of sites is the functionalized molecules, which can be protonated by an adsorbed hydrogen ion and carry one positive charge. At the same time, the protonation changes the dipole moment of the molecules, which will shift the potential of molecules additionally (As shown **Figure S10**(a)). The protonation of functionalized molecules follows the process below:

$$A + H_3O^+ \xleftrightarrow{K_c} A-H^+ + H_2O$$

Where, A represents the functionalized molecule. And the equivalent circuit can be simplified as the lift part of **Figure S10**(b). The molecules protonate-deprotonate with H^+ in electrolyte and build up an electric double layer, which represents as a capacitor C_{EDL2}. On the other hand, the dipole, induced by the protonation, shifts the potential of C_{EDL2} from φ_0 to φ_m. The equilibrium of the molecules can be described by:

$$\sigma_m = qN_m \frac{[H^+]exp(\frac{\varphi_{ea}}{kT})K_C}{1+K_C} \quad \text{(S-3)}$$

$$\varphi_{em} = \frac{2kT}{q} arcsin\,h(\frac{\sigma_{dm}}{\sqrt{8\varepsilon_0\varepsilon_w kTN_A c}}) \quad \text{(S-4)}$$

$$\varphi_{mo} = \frac{D([H^+])N_m}{\varepsilon_0\varepsilon_w} \quad \text{(S-5)}$$

Where, N_m is the functionalized molecule density, K_c is the reaction equilibrium constant of molecule protonation, D is the dipole moment of molecule, which varies with the hydrogen ion concentration.

The neutral condition of the system raises the requirement on charges:

$$\sigma_0 + \sigma_d + \sigma_{dm} + \sigma_m = 0 \quad \text{(S-6)}$$

For given pH, ion concentration of electrolyte, c, dipole moment at chosen pH, D, sites density of uncovered SiO_2, N_s, and functionalized molecule density, N_m, the potential and charge density at different position of the system can be calculated by solving the Eq. (S1-S6). We solve this equation group in MATLAB by *fsolve* function with *Levenberg-Marquardt* algorithm. The parameters used in the model and corresponding origins are listed in the **Table S2**.

Table S2. The parameters used in our model.

Parameter	Unit	Value	Origin(s)
k	$[J{\cdot}K^{-1}]$	1.38×10^{-23}	
T	[K]	300	
q	[C]	1.6×10^{-19}	
N_A	$[mol^{-1}]$	6.02×10^{23}	
ε_0	$[F{\cdot}m^{-1}]$	8.85×10^{-12}	
ε_w	-	80	
c	$[mol{\cdot}m^{-3}]$	1	Experiments
K_a	$[mol{\cdot}L^{-1}]$	1.5×10^{-2}	Ref[14]
K_b	$[mol{\cdot}L^{-1}]$	6×10^{-7}	Ref[14]
K_c		10	Fitting (constant)
C_{stern}	$[F{\cdot}m^{-2}]$	0.2	Ref[14]
N_s	$[m^{-2}]$	5×10^{15}~5×10^{17}	Fitting (changeable), Ref[15]
N_m	$[m^{-2}]$	1×10^{17}~5×10^{18}	Fitting (changeable)
D	[Debye]	-5.61~11	First principle calculation, optical absorption spectra
pH	-	1-7	Experiments

To fit the experimental results, ONLY two parameters are adjusted among different molecules: the molecule density N_m and the uncovered SiO_2 site density N_s. The dipole moment D of the molecule in protonated/unprotonated state are simulated by first principle calculation, and their variations according to pH follow the changing of character peaks in their optical absorption spectra (Figure 1b in the main text).

5. Synthetic procedures and characterization

All chemicals were purchased from Acros, Alfa Aser, Sigma Aldrich and TCI, respectively and used directly without further purification. All reactions were carried out by Schlenk ware technique under nitrogen. ^1H and ^{13}C NMR spectra were recorded with Bruker AMX 500, chemical shifts (δ)) are reported in ppm relative to the methine singlet for $CDCl_3$ (7.24, 77) and DMSO-*d*6 (2.50, 39.5). High-resolution molecular weight was determined by electrospray ionization mass (HR ESI) with dual ionization ESCi® (ESI/APCi) source options, Waters LCT Premier XE (Waters Corp., Manchester, UK), or MALDI mass spectrometry with DE-PRO mass spectrometer (Applied Biosystem).

Synthesis

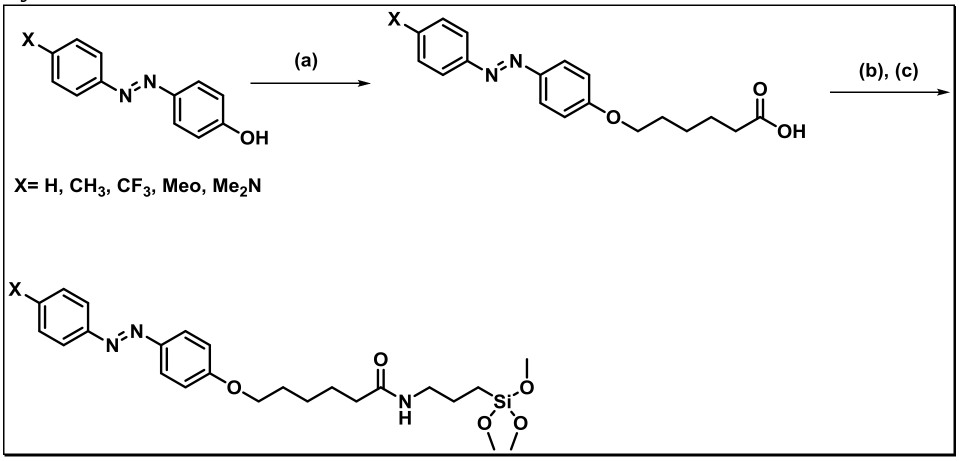

Scheme. (a) $BrC_5COOTHP$, K_2CO_3, KI, acetone, reflux then HCl/THF; (b) NHS, EDCI, DMF; then APTMS, CH_2Cl_2.

The synthesis procedures of these Azobenezene trimethoxysilane derivatives were prepared by a modified condition according to the previous method.[1] All of the preparation of the azobenezene trimethoxysilane derivatives in this article were followed with the procedure of methoxy-substituted one.

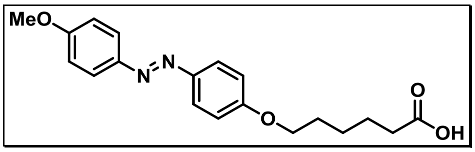

MeO-Azo-C_5COOH

A solution of 4-((4-methoxyphenyl)diazenyl)phenol (10 g, 43.8 mmol), potassium carbonate (11.08 g, 80 mmol), tetrahydro-2H-pyran-2-yl 6-bromohexanoate (18.35 g, 65.7 mmol) and potassium iodide (catalyst amount) in acetone solution was refluxed under nitrogen overnight. After cooling to room temperature, the solution was filtered, and the filtrate was concentrated. The residue was added a solution of tetrahydrofuran (300 mL) mixed with 12 N hydrogen chloride (60 mL) and stirred for 2 hours. The solution was poured into ice water and the solid was formed. The precipitate was filtered and washed with water. The filter cake was purified by recrystallization with dichloromethane/hexane to give the target product as yellow solid. Yield is 78 %.

^1H NMR (500 MHz, $CDCl_3$, δ): 7.86-7.83 (m, 4H, Ar H), 6.99-6.95 (m, 4H, Ar H), 4.02 (t, J = 6.4 Hz, 2H; OCH_2), 3.86 (s, 3H, OCH_3), 2.40 (t, J = 7.4 Hz, 2H; CH_2CO), 1.86-1.80 (m, 2H, CH_2), 1.76-1.69 (m, 2H, CH_2), 1.58-1.52 (m, 2H, CH_2); ^{13}C NMR (125M Hz, $CDCl_3$, δ): 177.36 (C=O), 161.54, 161.06, 147.11, 147.00, 124.34, 114.66, 114.17, 67.91, 55.56, 33.51,

28.89, 25.58, 24.44; HRMS (ESI) m/z: [M-H]$^+$ calcd for $C_{19}H_{21}N_2O_4$, 341.1501; found, 341.1500.

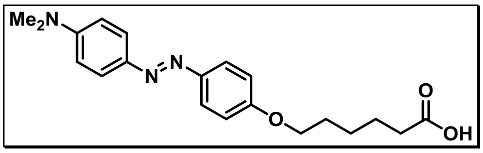

DMA-Azo-C_5COOH

^{1}H NMR (500 MHz, DMSO-*d6*, δ): 7.76-7.73 (m, 4H, Ar H), 7.06- 7.04 (m, 2H, Ar H), 6.83-6.81 (m, 2H, Ar H), 4.04 (t, *J* = 6.5 Hz, 2H; OCH_2), 3.04 (s, 6H, NCH_3), 2.24 (t, *J* = 7.3 Hz, 2H; CH_2CO), 1.77-1.72 (m, 2H, CH_2), 1.61- 1.55 (m, 2H, CH_2), 1.47-1.41 (m, 2H, CH_2); ^{13}C NMR (125 MHz, DMSO-*d6*, δ): 174.40 (C=O), 160.00, 152.02, 146.43, 142.57, 124.17, 123.46, 114.80, 111.56, 67.69, 33.60, 28.37, 25.10, 24.24; HRMS (ESI) m/z: [M+H]$^+$ calcd for $C_{20}H_{26}N_3O_3$, 356.1974; found: 356.1968.

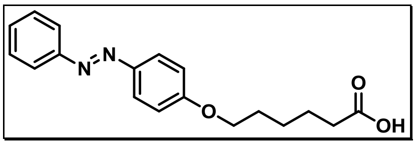

H-Azo-C_5COOH

^{1}H NMR (500 MHz, $CDCl_3$, δ): 7.90-7.85 (m, 4H, Ar H), 7.49-7.42 (m, 3H, Ar H), 6.99 6.97 (d, *J* = 8.9 Hz, 2H; Ar H), 4.05-4.02 (t, *J* = 6.4 Hz, 2H; OCH_2), 2.41 (t, *J* = 7.4 Hz, 2H; CH_2CO), 1.87-1.81 (m, 2H, CH_2), 1.76-1.70 (m, 2H, CH_2), 1.58-1.53 (m, 2H, CH_2); ^{13}C NMR (500 MHz, $CDCl_3$, δ): 178.7 (C=O), 161.8, 153.0, 147.2, 130.5, 129.3, 125.0, 122.8, 114.9, 68.2, 33.9, 29.1, 25.8, 24.6; HRMS (MALDI) m/z: [M+H]$^+$ calcd for $C_{18}H_{21}N_2O_3$, 313.1552; found 313.1564.

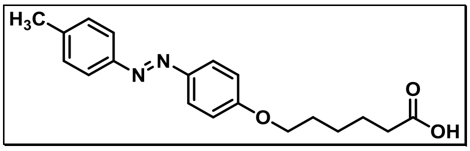

CH_3-Azo-C_5COOH

^{1}H NMR (500 MHz, $CDCl_3$, δ): 7.88 (d, *J* = 8.9 Hz, 2H; Ar H), 7.77 (d, *J* = 8.5 Hz, 2H; Ar H), 7.28 (d, *J* = 8.5 Hz, 2H; Ar H), 6.97 (d, *J* = 8.9 Hz, 2H; Ar H), 4.04 (t, *J* = 6.4 Hz, 2H; OCH_2), 2.42-2.39 (m, 5H; CH_2CO, CH_3), 1.86-1.81 (m, 2H, CH_2), 1.75-1.69 (m, 2H, CH_2), 1.58-1.53 (m, 2H, CH_2); ^{13}C NMR (500 MHz, $CDCl_3$, δ): 177.8 (C=O), 161.6, 151.0, 147.1, 141.0, 129.9, 124.9, 122.8, 114.9, 68.2, 33.8, 29.1, 25.8, 24.7, 21.7; HRMS (MALDI) m/z: [M+Na]$^+$ calcd for $C_{19}H_{22}N_2O_3Na$, 349.1528; found 349.1540.

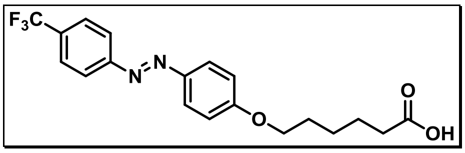

CF_3-Azo-C_5COOH

^{1}H NMR (500 MHz, $CDCl_3$, δ): 7.94-7.91 (m, 4H; Ar H), 7.74 (d, *J* = 8.6 Hz, 2H; Ar H), 6.99 (d, *J* = 8.9 Hz, 2H; Ar H), 4.04 (t, *J* = 6.4 Hz, 2H; OCH_2), 2.40 (t, *J* = 7.4 Hz, 2H; CH_2CO), 1.86-1.83 (m, 2H, CH_2), 1.75-1.70 (m, 2H, CH_2), 1.58-1.52 (m, 2H, CH_2); ^{13}C NMR (500 MHz, $CDCl_3$, δ): 177.1 (C=O), 162.5, 155.3, 147.1, 131.9, 126.5, 126.4, 125.5, 122.9, 115.1, 68.3, 33.6, 29.1, 25.8, 24.6; HRMS (MALDI) m/z: [M+Na]$^+$ calcd for $C_{19}H_{19}F_3N_2O_3Na$, 403.1246; found 403.1252.

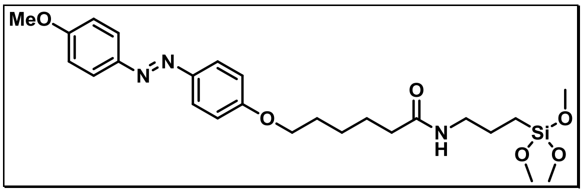

MeO-Azo trimethoxysilane

6-(4-((4-methoxyphenyl)diazenyl)phenoxy)hexanoic acid, MeO-Azo-C_5COOH, (4.0 g, 11.7 mmol), N-hydroxysuccinimide, NHS (3.5 g, 30.4 mmol), N-(3-dimethylaminopropyl)-N'-ethylcarbodiimide hydrochloride, EDCI (4.5 g, 23.4 mmol) and dry N,N-dimethylforamide (70 mL) were all placed in an oven-dried flask and stirred overnight. The solution was poured into ice water and filtered. The filter cake was washed with water and dried without further purification to undergo next step. A dry dichloromethane solution (120 mL) of N-hydroxysuccinimide substituted derivative (2.5 g, 5.7 mmol) was added (3-aminopropyl)trimethoxysilane (3.1 g, 17.1 mmol) and stirred under nitrogen for three hours. After the starting material was completely finished, the organic solution was washed with water three times, dried by anhydrous magnesium sulfate and concentrated. The crude was purified by recrystallization with dichloromethane/hexane to give target product as yellow solid. Yield is 58%.

^{1}H NMR (500 MHz, $CDCl_3$, δ): 7.86-7.83 (m, 4H, Ar H), 6.99-6.94 (m, 4H, Ar H), 5.67 (s, 1H; CONH), 4.0 (t, *J* = 6.4 Hz, 2H; OCH_2), 3.85 (s, 3H; OCH_3), 3.54 (s, 9H; $SiOCH_3$), 3.22 (td, *J* = 6.4, 6.6 Hz, 2H; CH_2), 2.17 (t, *J* = 7.5 Hz, 2H; $COCH_2$), 1.83-1.78 (m, 2H; CH_2), 1.73-1.66 (m, 2H; CH_2), 1.62-1.56 (m, 2H; CH_2), 1.52-1.46 (m, 2H; CH_2), 0.62 (t, *J* = 8.1 Hz, 2H; $SiCH_2$); ^{13}C NMR (125 MHz, $CDCl_3$, δ): 172.70 (C=O), 161.52, 161.06, 147.09, 146.95, 124.32, 114.63, 114.14, 67.96, 55.53, 50.59, 41.75, 36.71, 28.96, 25.78, 25.44, 22.75, 6.48; HRMS (ESI) m/z: $[M+Na]^+$ calcd for $C_{25}H_{37}N_3O_6SiNa$, 526.2349; found: 526.2354.

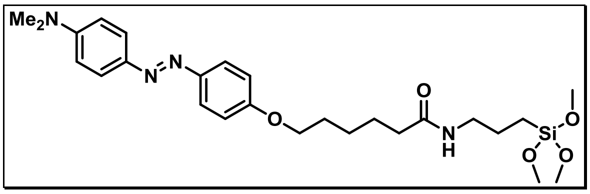

DMA-Azo trimethoxysilane

^{1}H NMR (500 MHz, $CDCl_3$, δ): 7.82-7.79 (m, 4H, Ar H), 6.94 (d, *J* = 9.0 Hz, 2H; Ar H), 6.73 (d, *J* = 9.1 Hz, 2H; Ar H), 5.68 (s, 1H; CONH), 4.00 (t, *J* = 6.5 Hz, 2H; OCH_2), 3.55 (s, 9H; $SiOCH_3$), 3.22 (td, *J* = 6.2, 6.6 Hz, 2H; NCH_2), 3.04 (s, 6H; ; NCH_3), 2.17 (t, *J* = 7.5 Hz, 2H; $COCH_2$), 1.83-1.78 (m, 2H, CH_2), 1.73-1.67 (m, 2H, CH_2), 1.63-1.57 (m, 2H, CH_2), 1.53-1.47 (m, 2H, CH_2), 0.63 (t, *J* = 8.1 Hz, 2H; $SiCH_2$); ^{13}C NMR (125 MHz, $CDCl_3$, δ): 172.73 (C=O), 160.32, 152.00, 147.35, 143.69, 124.45, 123.80, 114.57, 111.57, 67.90, 50.59, 41.75, 40.32, 36.72, 28.99, 25.79, 25.46, 22.75, 6.47; HRMS (ESI) m/z: $[M+Na]^+$ calcd for $C_{26}H_{40}N_4O_5SiNa$, 539.2666; found: 539.2661.

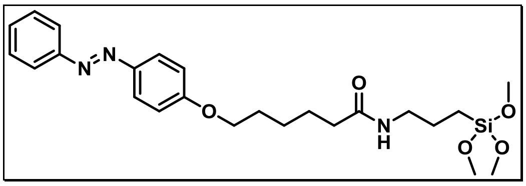

H-Azo trimethoxysilane

^{1}H NMR (500 MHz, $CDCl_3$, δ): 7.89-7.84 (m, 4H, Ar H), 7.49-7.41 (m, 3H, Ar H), 6.97 (d, *J* = 9.0 Hz, 2H; Ar H), 5.65 (s, br, 1H; CONH), 4.02 (t, *J* = 6.4 Hz, 2H; OCH_2), 3.55 (s, 9H, $SiOCH_3$), 3.26-3.22 (m, 2H; NCH_2), 2.18 (t, *J* = 7.6 Hz, 2H, $COCH_2$), 1.86-1.49 (m, 8H, CH_2), 0.63 (t, *J* = 8.1 Hz, 2H; $SiCH_2$); ^{13}C NMR (125 MHz, $CDCl_3$, δ): 172.9 (C=O), 161.8, 153.1, 147.2, 130.5, 129.2, 125.0, 122.8, 114.9, 68.3, 50.8, 42.0, 36.9, 29.2, 26.0, 25.7, 23.0, 6.8; HRMS (MALDI) m/z: $[M+Na]^+$ calcd for $C_{24}H_{35}N_3O_5SiNa$, 496.2244; found 496.2264.

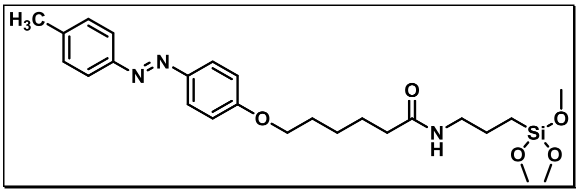

CH_3-Azo trimethoxysilane

^{1}H NMR (500 MHz, $CDCl_3$, δ): 7.86 (d, *J* = 8.9 Hz, 2H; Ar H), 7.77 (d, *J* = 8.4 Hz, 2H; Ar H), 7.27 (d, *J* = 8.1 Hz, 2H; Ar H), 6.97 (d, *J* = 8.9 Hz, 2H; Ar H), 5.66 (s, br, 1H; CONH), 4.02 (t, *J* = 6.4 Hz, 2H; OCH_2), 3.55 (s, 9H, $SiOCH_3$), 3.25-3.22 (m, 2H, NCH_2), 2.40 (s, 3H, $ArCH_3$), 2.17 (t, *J* = 7.5 Hz, 2H; $COCH_2$), 1.84-1.49 (m, 8H, CH_2), 0.63 (t, *J* = 8.1 Hz, 2H; $SiCH_2$); ^{13}C NMR (125 MHz, $CDCl_3$, δ): 172.9 (C=O), 161.6, 151.1, 147.2, 141.0, 129.9, 124.8, 122.7, 114.9, 68.2, 50.8, 42.0, 36.9, 29.2, 26.0, 25.7, 23.0, 21.6, 6.7; HRMS (MALDI) m/z: $[M+Na]^+$ calcd for $C_{25}H_{37}N_3O_5SiNa$, 510.2400; found 510.2424.

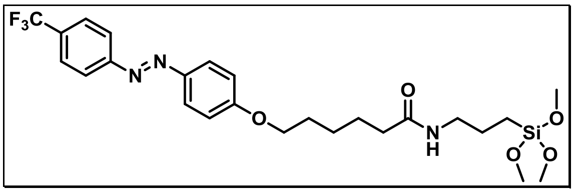

CF_3-Azo trimethoxysilane

^{1}H NMR (500 MHz, $CDCl_3$, δ): 7.94-7.91 (m, 4H, Ar H), 7.73 (d, *J* = 8.4 Hz, 2H, Ar H), 6.99 (d, *J* =9.0 Hz, 2H, Ar H), 5.65 (s, br, 1H, CONH), 4.02 (t, *J* = 6.4 Hz, 2H; OCH_2), 3.52 (s, 9H, $SiOCH_3$), 3.26-3.21 (m, 2H, NCH_2), 2.20 (t, *J* = 7.5 Hz, 2H, $COCH_2$), 1.83-1.49 (m, 8H, CH_2), 0.63(t, *J* =8.1 Hz, 2H; CH_2Si); ^{13}C NMR (125M Hz, $CDCl_3$, δ): 172.9 (C=O), 162.5, 154.9, 147.0, 131.9, 126.4(2), 125.4, 122.9, 115.0, 68.4, 50.8, 42.0, 36.9, 29.2, 26.0, 25.6, 23.0, 6.8; HRMS (MALDI) m/z: $[M+Na]^+$ calcd for $C_{25}H_{34}F_3N_3O_5SiNa$, 564.2117; found 564.2137.

References

[1] C. W. Tseng, D. C. Huang, Y. T. Tao, *ACS Appl. Mater. Interfaces.* **2012**, *4*, 5483.
[2] G. Demirel, M. O. Caglayan, B. Garipcan, *Nanoscale Res. Lett.* **2007**, *2*, 350-354.
[3] S. Y. Song, R. Q. Chu, J. F. Zhou, S. R. Yang, J. Y. Zhang, *J. Phys. Chem. C* **2008**, 112, 3805-3810.
[4] S. Chen, N. Jokilaakso, P. Björk, A. Eriksson Karlström, S.-L. Zhang, *Applied Physics Letters* **2010**, *97*, 264102.
[5] Z. Zhang, S.-L. Zhang, M. Östling, J. Lu, *Appl. Phys. Lett.* **2006**, *88*, 142114.
[6] S. Chen, S.-L. Zhang, *Journal of Vacuum Science & Technology A* **2011**, *29*, 011022.
[7] Y. Liu, P. Georgiou, T. Prodromakis, T. G. Constandinou, C. Toumazou, *IEEE Trans. Electron Devices* **2011**, *58*, 4414-4422.
[8] (a) A. D. Becke, *J. Chem. Phys.* **1993**, *98*, 5648. (b) C. Lee, W. Yang, R. G. Parr, *Phys. Rev. B* **1988**, *37*, 785.
[9] S. Grimme, *J. Comp. Chem.* **2006**, *27*, 1787.
[10] (a) D. Feller, *J. Comp. Chem.* **1996**, *17*, 1571. (b) K. L. Schuchardt, B. T. Didier, T. Elsethagen, L. Sun, V. Gurumoorthi, J. Chase, J. Li, T. L. Windus, *J. Chem. Inf. Model.* **2007**, *47*, 1045.
[11] M. J. Frisch, *GAUSSIAN 09, Revision E. 01*, Gaussian Inc., Wallingford CT, **2009**.
[12] R. E. G. van Hal, J. C. T. Eijkel, P. Bergveld, *Advances in Colloid and Interface Science* **1996**, *69*, 31-62.
[13] A. J. Bard, L. R. Faulkner, *Electrochemical Methods, Fundamentals and Applications,* 2nd ed. New York: John Wiley & Sons, Inc., **2001**.
[14] M. Grattarola, G. Massobrio, S. Martinoia, *IEEE Trans. Electron Devices* **1992**, *39*, 813-819.
[15] R.E.G. van Hal, J.C.T. Eijkel, P. Bergveld. *Sensors and Actuators B* **1995**, *24-25*, 201-205.
